# Supplementary material for: Circadian rhythms of macrophages are altered by the acidic tumor microenvironment
Source: EMBO Rep. 2024 Oct 16;25(11):23. doi: 10.1038/s44319-024-00288-2 (PMC11549407; doi:10.1038/s44319-024-00288-2)
Supplement: Supplementary file 1 — Appendix [file 44319_2024_288_MOESM1_ESM.pdf]

Appendix for:

Circadian rhythms of macrophages are altered by the acidic tumor microenvironment

All correspondence: Brian J. Altman

E-mail: Brian_Altman@URMC.rochester.edu

Table of Contents

Face Page and Table of Contents……Page 1

Appendix Figure S1…………………….Page 2

Appendix Figure S2…………………….Page 3

Appendix Figure S3…………………….Page 4

Appendix Figure S4…………………….Page 5

Appendix Table S1…………………….Page 6

Appendix Figure S1

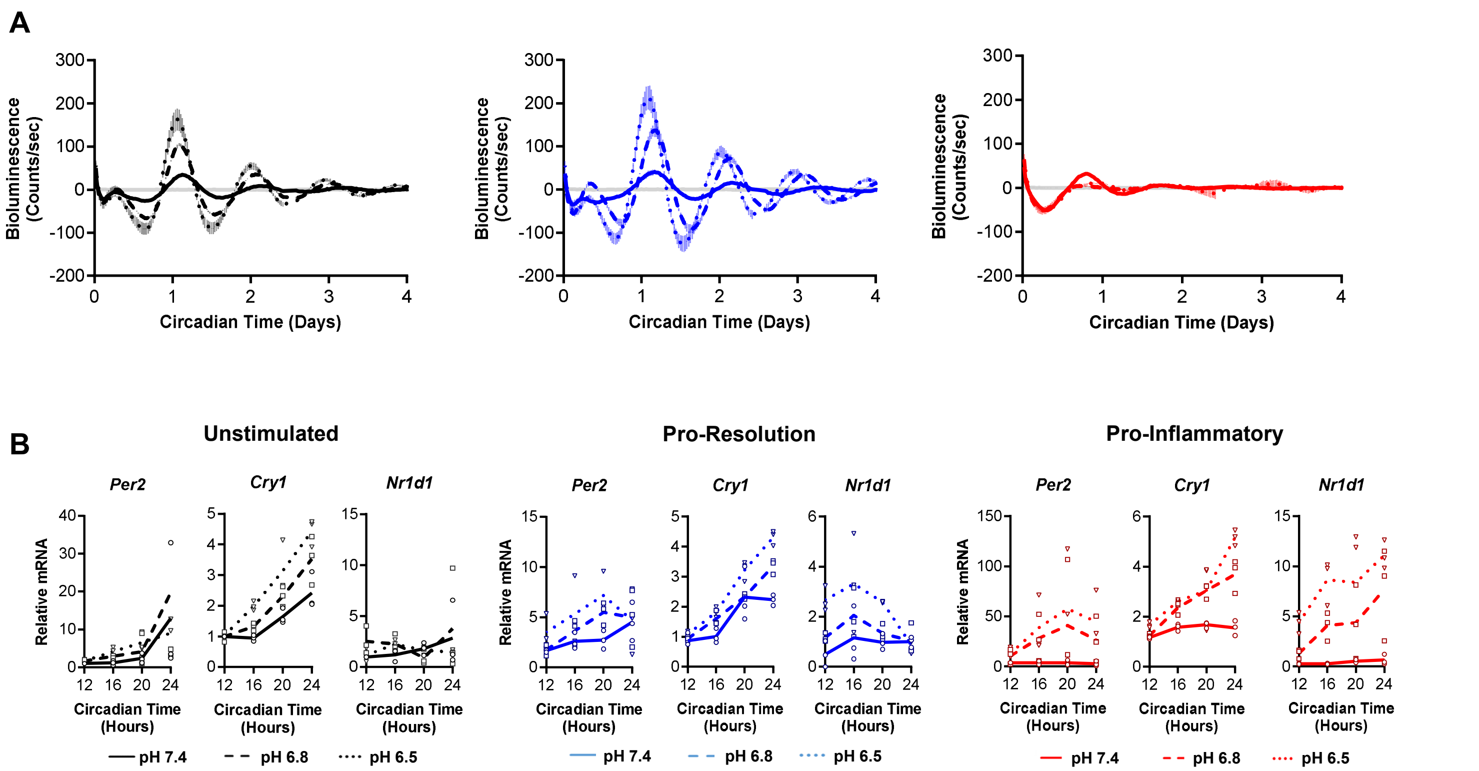

Appendix Figure S1. Axis-matched and individual point data for Figure 2

A. Lumicycle data from **Figure 2A-C** are presented as axis-matched graphs.

B. Individual data points and mean are displayed for data presented in **Figure 2D-F**.

Data information: For (**A**), shown are the mean and SEM. For (B), shown are individual data points and the mean.

Appendix Figure S2

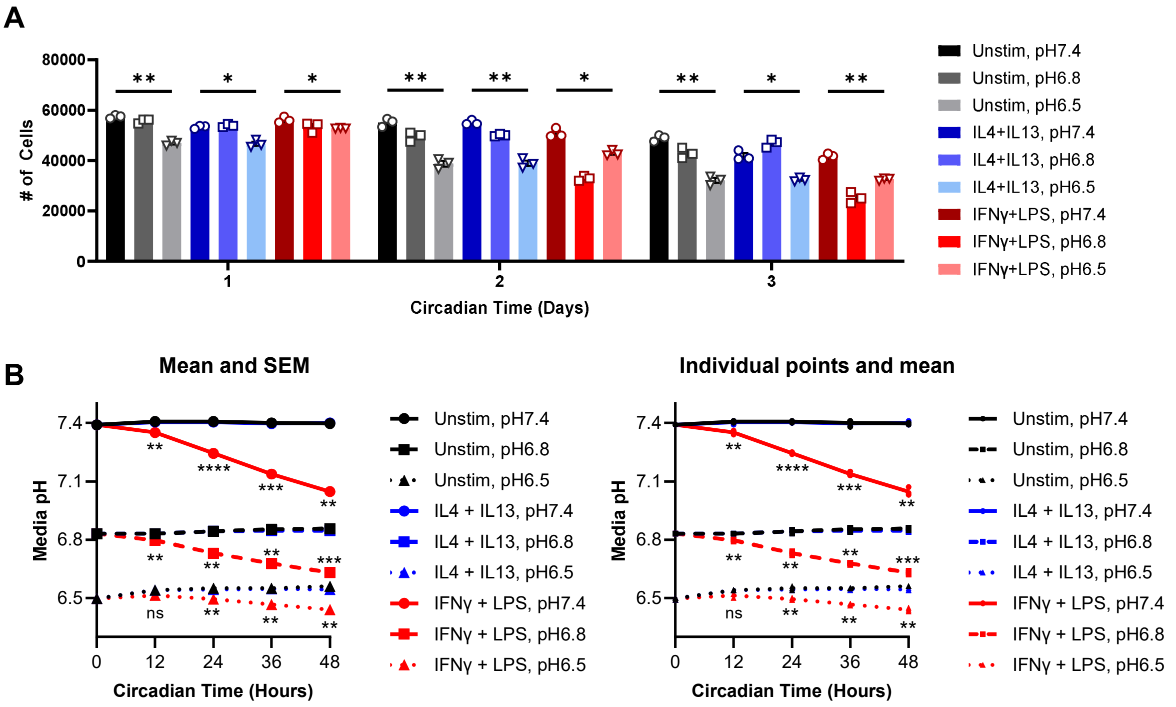

Appendix Figure S2. Survival of macrophages under acidic pH, and acidification of media by macrophages.

A. Bone marrow-derived macrophages (BMDMs) were obtained from C57BL/6 mice expressing Per2-Luc. The circadian clocks of BMDMs were synchronized by a 24-hour period of serum starvation in media with 0% serum, followed by a 2-hour period of serum shock in media with 50% serum. BMDMs were then cultured in media with pH 7.4 or acidic media with pH 6.8 or 6.5, and stimulated with either 10 ng/mL IL-4 and 10 ng/mL IL-13, or 50 ng/mL IFNγ and 100 ng/mL LPS; or left unstimulated. Cells were fixed at CT 1, 2, and 3 days post-treatment and stained with DAPI. Number of nuclei was counted using Celigo to determine the number of adherent cells, n=3 biological replicates.

B. Supernatant from **Figure 2A** was collected at CT 12, 24, 36, and 48 hours and pH of media was measured, n=3 biological replicates. Left panel is mean and SEM, right panel is individual points and mean.

Data information: For (**A B** left panel), shown are the mean and SEM. Statistical significance determined by multiple unpaired t-test with Welch's correction. The Holm- Šídák correction for multiple t-tests was applied. For (**A**), pH 7.4 was compared to pH 6.5 only. For (**B**), unstimulated was compared to IFNγ + LPS (pro-Inflammatory) only; *, $p < 0.05$; **, $p<0.005$; ***, $p<0.0005$; ****, $p<0.0001$; ns: not significant. All experiments were replicated twice. Exact p values: **B.** Unstimulated pH 7.4 vs pH 6.5 0.00058 (Day 1), 0.000629 (Day 2), 0.000629 (Day 3); IL4 + IL13 (pro-Resolution) pH 7.4 vs pH 6.5 0.013315 (Day 1), 0.002528 (Day 2), 0.013315 (Day 3); IFNγ + LPS (pro-Inflammatory) pH 7.4 vs pH 6.5 0.041542 (Day 1), 0.011097 (Day 2), 0.008171 (Day 3). **C.** Unstimulated vs IFNγ + LPS (pro-Inflammatory) starting pH 7.4 0.002665 (CT12), <0.0001 (CT24), 0.000222 (CT36), 0.001131 (CT48); Unstimulated vs IFNγ + LPS (pro-Inflammatory) starting pH 6.8 0.009852 (CT12), 0.000564 (CT24), 0.000564 (CT36), 0.000133 (CT48); unstimulated vs IFNγ + LPS (pro-Inflammatory) starting pH 6.5 0.009195 (CT24), 0.007955 (CT36), 0.009195 (CT48).

Appendix Figure S3

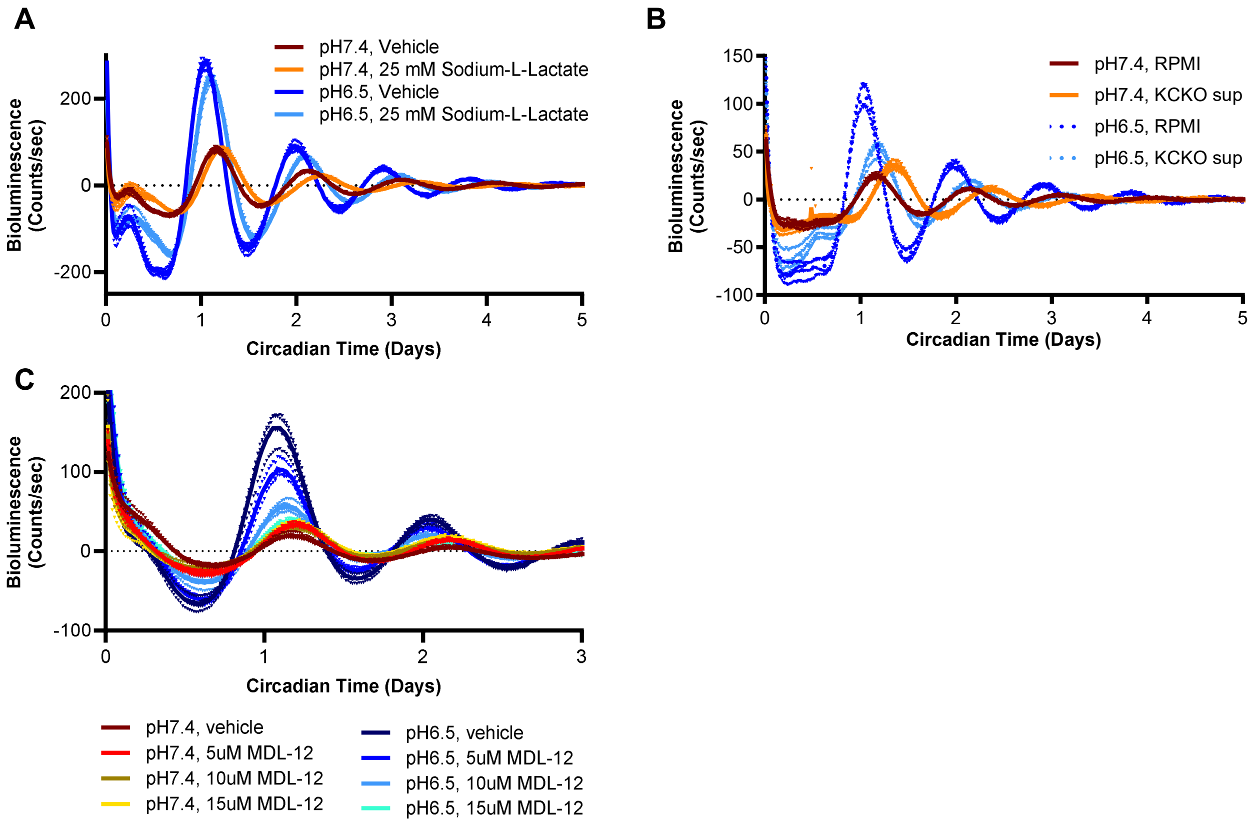

Appendix Figure S3. Individual data points from Figures 4, 5, EV3.

A. Individual data points and mean are displayed for data presented in **Figure 4B**.

B. Individual data points and mean are displayed for data presented in **Figure EV3.**

C. Individual data points and mean are displayed for data presented in **Figure 5C.**

Appendix Figure S4

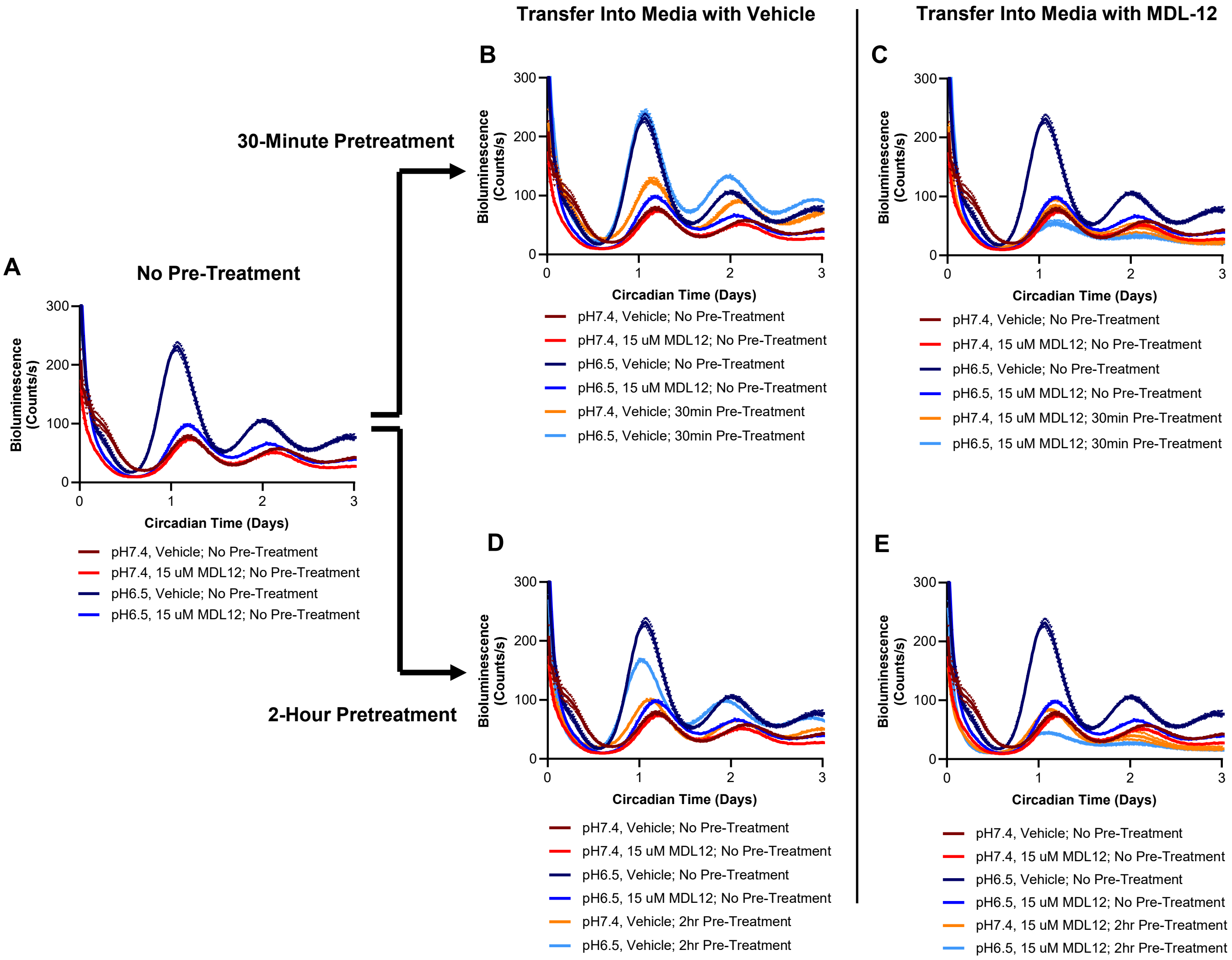

Appendix Figure S4. Pre-treatment with 15 uM MDL-12 for up to 2 hours is not necessary to suppress pH-driven changes in amplitude of circadian rhythms.

A-E. Bone marrow-derived macrophages (BMDMs) were obtained from C57BL/6 mice expressing PER2-Luc. The circadian clocks of BMDMs were synchronized by a 24-hour period of serum starvation in media with 0% serum, followed by a 2-hour period of serum shock in media with 50% serum. (**A**) BMDMs were then cultured in media with neutral pH 7.4 or acidic pH 6.5, and treated with vehicle or 15uM MDL-12. Alternately, (**B,C**) BMDMs were cultured at pH 7.4 and pre-treated with 15uM MDL-12 for 30 minutes or (**D,E**) 2 hours, after which media was removed and cells were cultured in media at pH 7.4 or pH 6.5 in the (**C,E**) presence or (**B,D**) absence of MDL-12. Luciferase activity was monitored in real time by LumiCycle. For each curve, n=2 biological replicates. Data for cells that received no pre-treatment (**A**) was overlaid on plots of data where cells received pre-treatment (**B-E**) to allow for comparison of changes in rhythms.

Data information: Shown are individual points and mean.

Appendix Table S1

A

	p_val	avg_log2FC	pct.1	pct.2	p_val_adj	cluster	gene
**Bhlhe40**	2.87E-69	1.310254571	0.705	0.436	4.10E-65	1	Bhlhe40
**Nfil3**	3.37E-18	0.675795838	0.576	0.46	4.82E-14	1	Nfil3
**Bhlhe41**	6.52E-17	1.616565895	0.153	0.067	9.32E-13	1	Bhlhe41
**Bhlhe40.1**	5.07E-32	-1.315329841	0.312	0.515	7.26E-28	2	Bhlhe40
**Rora.1**	5.02E-13	-2.374329382	0.025	0.108	7.17E-09	2	Rora
**Bhlhe41.1**	2.80E-54	1.66383049	0.241	0.057	4.00E-50	3	Bhlhe41
**Nfil3.2**	3.59E-22	-1.03184966	0.315	0.505	5.13E-18	3	Nfil3
**Bhlhe40.2**	1.97E-10	-0.793298973	0.389	0.496	2.81622E-06	3	Bhlhe40
**Rora.3**	9.60E-75	2.359976626	0.326	0.069	1.37E-70	4	Rora
**Crem.2**	3.75E-28	1.115605774	0.423	0.203	5.36E-24	4	Crem
**Bhlhe40.3**	1.22E-31	0.818941123	0.715	0.456	1.75E-27	5	Bhlhe40
**Nfil3.4**	3.26E-22	0.728660816	0.66	0.46	4.66E-18	5	Nfil3
**Nfil3.5**	1.23E-27	0.950463237	0.669	0.459	1.76E-23	6	Nfil3
**Bhlhe40.5**	3.41E-07	-0.673485523	0.377	0.492	0.004869931	7	Bhlhe40
**Bhlhe40.6**	2.73E-50	-3.204906923	0.114	0.514	3.91E-46	8	Bhlhe40
**Nfil3.6**	7.58E-37	-2.098752349	0.167	0.506	1.08E-32	8	Nfil3
**Dbp**	1.25E-34	2.817971988	0.156	0.029	1.78E-30	8	Dbp
**Nr1d2**	1.09E-13	1.807245581	0.114	0.035	1.56E-09	8	Nr1d2
**Crem.5**	5.45E-09	-1.337453444	0.106	0.236	7.78806E-05	8	Crem

B

	p_val	avg_log2FC	pct.1	pct.2	p_val_adj	cluster	gene
**Crem**	1.90E-31	-0.981727363	0.18	0.332	2.71E-27	CREM-Low	Crem
**Nfil3**	1.20E-27	-0.641877469	0.434	0.587	1.71E-23	CREM-Low	Nfil3
**Rora**	3.99E-26	-1.418121171	0.065	0.163	5.71E-22	CREM-Low	Rora
**Bhlhe40**	1.27E-25	-0.475243791	0.434	0.596	1.82E-21	CREM-Low	Bhlhe40
**Cry1**	9.18E-07	-0.699260507	0.076	0.12	0.013122	CREM-Low	Cry1
**Crem.1**	1.90E-31	0.981727363	0.332	0.18	2.71E-27	CREM-High	Crem
**Nfil3.1**	1.20E-27	0.641877469	0.587	0.434	1.71E-23	CREM-High	Nfil3
**Rora.1**	3.99E-26	1.418121171	0.163	0.065	5.71E-22	CREM-High	Rora
**Bhlhe40.1**	1.27E-25	0.475243791	0.596	0.434	1.82E-21	CREM-High	Bhlhe40
**Cry1.1**	9.18E-07	0.699260507	0.12	0.076	0.013122	CREM-High	Cry1

Appendix Table S1. Complete exact p values for all scRNA-seq comparisons in Figure 9.

A. Exact p values for all comparisons in **Figure 9B.**

B. Exact p values for all comparisons in **Figures 9C,E.**
