# Supplementary material for: Circadian rhythms of macrophages are altered by the acidic tumor microenvironment
Source: EMBO Rep. 2024 Oct 16;25(11):23. doi: 10.1038/s44319-024-00288-2 (PMC11549407; doi:10.1038/s44319-024-00288-2)
Supplement: Supplementary file 3 — Expanded View Figures [file 44319_2024_288_MOESM3_ESM.pdf]

Expanded View Figures

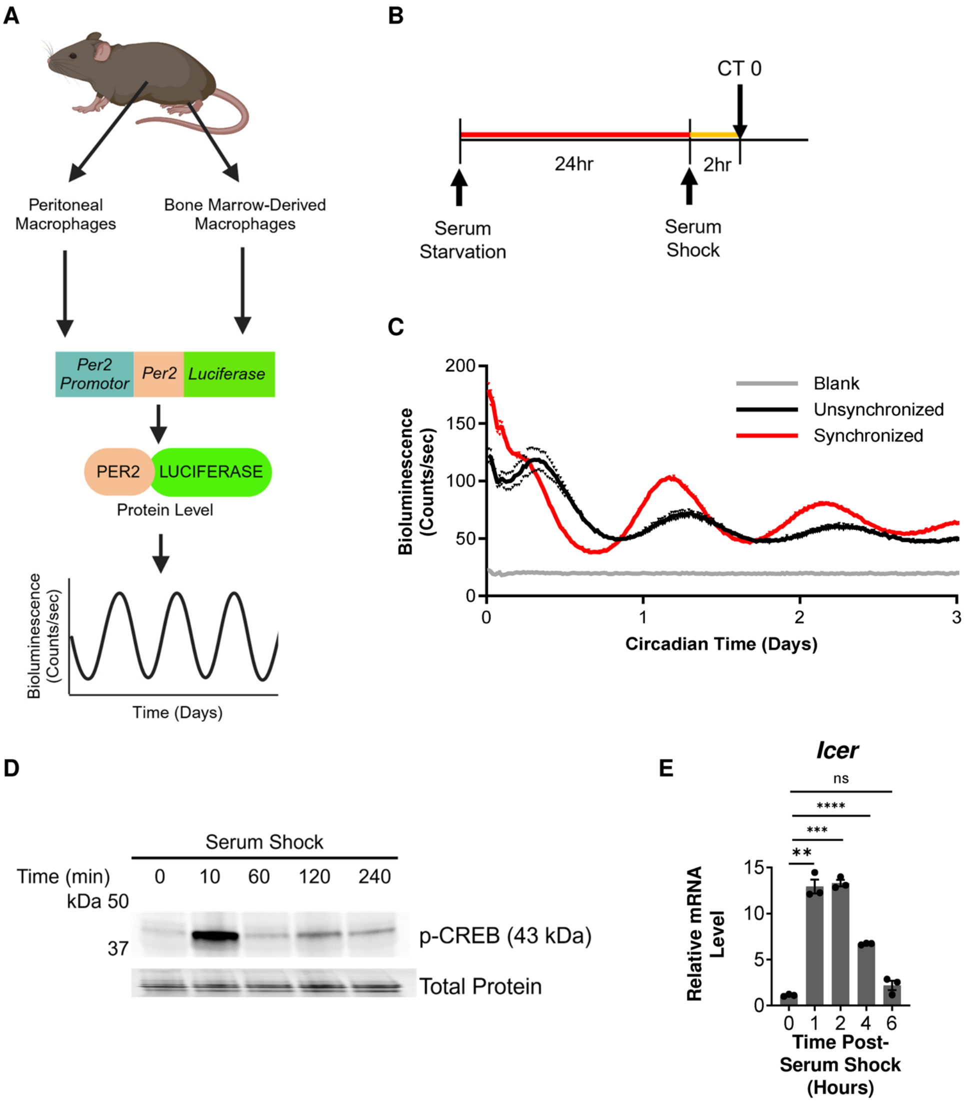

Figure EV1. The PER2-Luciferase reporter system enables real-time monitoring of circadian rhythms of macrophages.

(**A**) A schematic of the Per2-Luciferase (Per2-Luc) luciferase reporter system. (**B**) A schematic of the synchronization protocol in which the circadian clocks of bone marrow-derived macrophages (BMDMs) derived from C57BL/6 mice expressing Per2-Luc were synchronized by a 24-h period of serum starvation in media with 0% serum, followed by a 2-h period of serum shock in media with 50% serum. (**C**) BMDMs were then cultured in RPMI/10% FBS supplemented with D-luciferin at circadian time (CT) 0. Luciferase activity of BMDMs was monitored in real time by LumiCycle, $n = 2$ biological replicates. (**D**, **E**) Protein and RNA were collected at the indicated times post-serum shock to assess (**D**) cAMP signaling by p-CREB levels and (**E**) expression of *Icer*, $n = 3$ biological replicates. Data information: For (**C–E**), experiments were replicated twice. For (**C**), shown are individual points and mean. For (**E**), mean and SEM are shown, and statistical significance determined by unpaired two-tailed t-test with Welch's correction; $*p < 0.05$; $**p < 0.005$; $***p < 0.0005$; $****p < 0.0001$; ns: not significant. Exact *p* values: (**E**) 0.0039 (0 h vs 1 h), 0.0006 (0 h vs 2 h), <0.0001 (0 h vs 4 h).

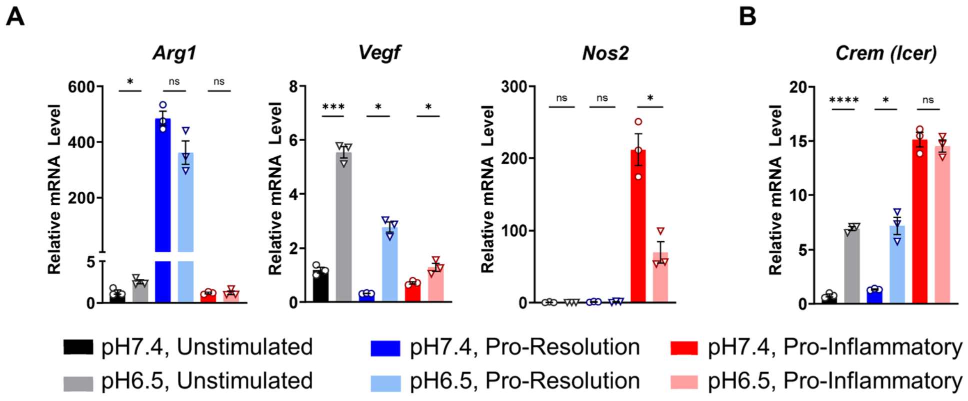

Figure EV2. Macrophages sense and respond to an acidic extracellular environment when cultured in vitro in media with acidic pH.

(**A**, **B**) Bone marrow-derived macrophages (BMDMs) were obtained from C57BL/6 mice expressing PER2-Luc. BMDMs were cultured in media with pH 7.4 or acidic media with pH 6.5, and stimulated with either 10 ng/mL IL-4 and 10 ng/mL IL-13 (pro-resolution), or 50 ng/mL IFNγ and 100 ng/mL LPS (pro-inflammatory); or left unstimulated. RNA was collected at 2 h post-treatment, and qt-PCR was performed to assess expression of genes associated with (**A**) phenotype or (**B**) acid sensing in macrophages. For both panels, $n = 3$ biological replicates. Data information: Shown are mean and SEM. Statistical significance determined by two-tailed t-test with Welch's correction. The Holm-Šídák correction for multiple t-tests were applied; *$p < 0.05$; **$p < 0.005$; ***$p < 0.0005$; ****$p < 0.0001$; ns: not significant. Experiments were replicated twice. Exact p values: (**A**) *Arg1* 0.024372 (pH 7.4 vs pH 6.5 unstimulated); *Vegf* 0.000403 (pH 7.4 vs pH 6.5 unstimulated), 0.011077 (pH 7.4 vs pH 6.5 pro-resolution), 0.048413 (pH 7.4 vs pH 6.5 pro-Inflammatory); *Nos2* 0.008509 (pH 7.4 vs pH 6.5 pro-inflammatory). (**B**) *Icer* < 0.0001 (pH 7.4 vs pH 6.5 unstimulated), 0.01749 (pH 7.4 vs pH 6.5 pro-resolution).

© The Author(s)

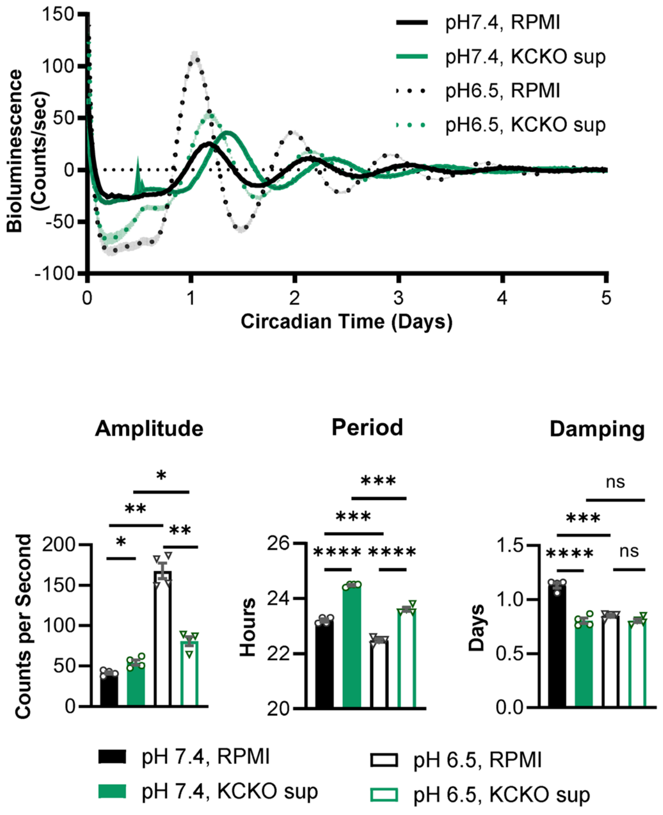

Figure EV3. Exposure to cancer cell supernatant further modulates circadian rhythms in addition to pH-driven changes.

Bone marrow-derived macrophages (BMDMs) were obtained from C57BL/6 mice expressing Per2-Luc. The circadian clocks of BMDMs were synchronized by a 24-h period of serum starvation in media with 0% serum, followed by a 2-h period of serum shock in media with 50% serum. BMDMs were then cultured in RPMI with neutral pH 7.4 or acidic pH 6.5, or in KCKO supernatant at pH 6.5 or pH-adjusted to pH 7.4. Luciferase activity was monitored in real time by LumiCycle. Data was baseline-subtracted using the running average, and oscillation parameters were measured by LumiCycle Analysis, $n = 4$ biological replicates. Data information: Shown is the mean and SEM. Statistical significance determined by unpaired two-tailed t-test with Welch's correction; $*p < 0.05$; $**p < 0.005$; $***p < 0.0005$; $****p < 0.0001$; ns: not significant. Exact p values: Amplitude 0.0216 (pH 7.4 RPMI vs pH 7.4 KCKO sup), 0.0008 (pH 7.4 RPMI vs pH 6.5 RPMI), 0.0105 (pH 7.4 KCKO sup vs pH 6.5 KCKO sup), 0.0007 (pH 6.5 RPMI vs pH 6.5 KCKO sup); Period <0.0001 (pH 7.4 RPMI vs pH 7.4 KCKO sup), 0.0002 (pH 7.4 RPMI vs pH 6.5 RPMI), 0.0004 (pH 7.4 KCKO sup vs pH 6.5 KCKO sup), <0.0001 (pH 6.5 RPMI vs pH 6.5 KCKO sup); Damping <0.0001 (pH 7.4 RPMI vs pH 7.4 KCKO sup), 0.0002 (pH 7.4 RPMI vs pH 6.5 RPMI).

© The Author(s)

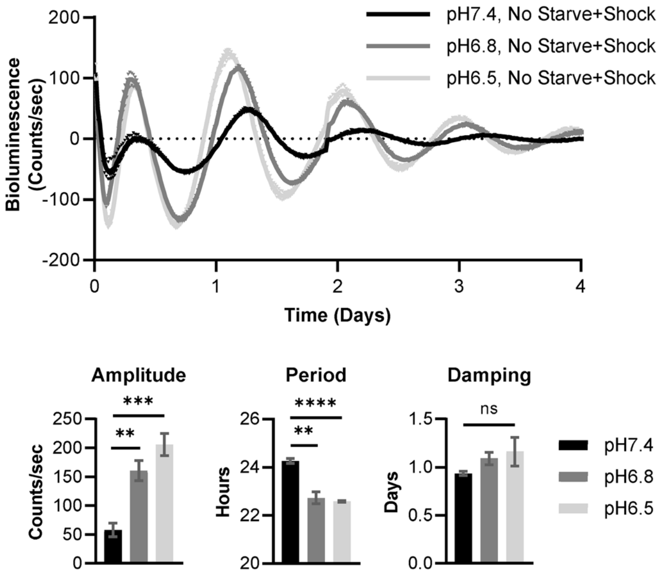

Figure EV4. Acidic pH alters circadian rhythms in macrophages in the absence of prior serum starvation followed by serum shock.

Bone marrow-derived macrophages (BMDMs) were obtained from C57BL/6 mice expressing Per2-Luc. BMDMs were cultured in media with neutral pH 7.4 or acidic pH 6.8 or 6.5. Luciferase activity was monitored in real time by LumiCycle (pH 7.4: $n = 3$. pH 6.8: $n = 2$. pH 6.5: $n = 3$). Data was baseline-subtracted using the running average, and oscillation parameters were measured by LumiCycle Analysis (pH 7.4: $n = 5$. pH 6.8: $n = 4$. pH 6.5: $n = 5$, data pooled from 2 individual experiments). Data information: For LumiCycle traces, shown are individual points and mean. For LumiCycle analysis, shown is the mean and SEM. Statistical significance determined by unpaired two-tailed t-test with Welch's correction (pH 6.8 and 6.5 comparison not tested); $^{**}p < 0.005$; $^{***}p < 0.0005$; $^{****}p < 0.0001$. Exact p values: Amplitude 0.0035 (pH 7.4 vs pH 6.8), 0.0004 (pH 7.4 vs pH 6.5); Period 0.0044 (pH 7.4 vs pH 6.8), <0.0001 (pH 7.4 vs pH 6.5).

 © The Author(s)

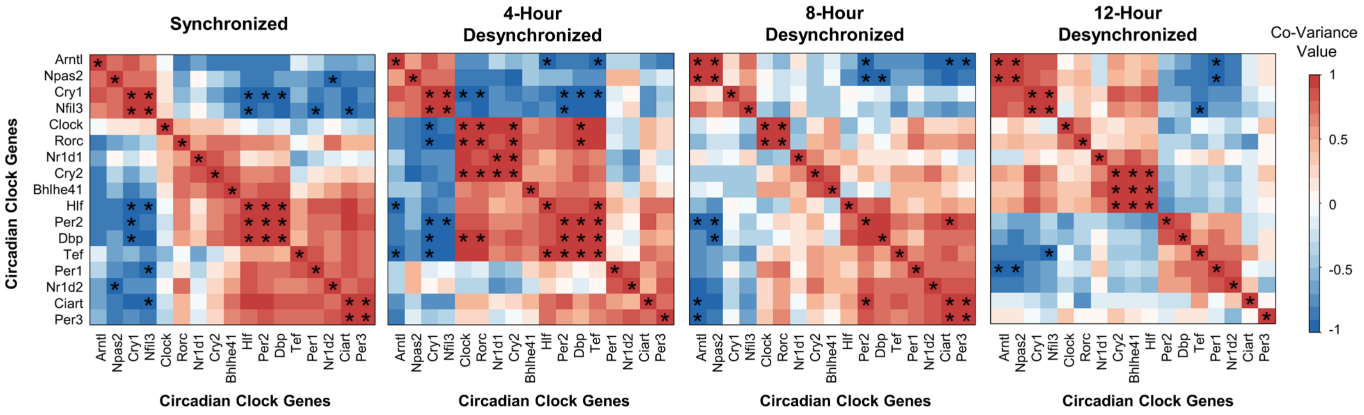

Figure EV5. Heterogeneity in circadian rhythms of cells within a population can lead to an altered circadian clock gene network in samples.

Increasingly desynchronized populations were modeled using an RNA-seq data set of WT peritoneal macrophages taken at 4-h intervals across two days, $n = 12$ biological replicates (see Fig. 8 and Methods). Weighted gene co-expression network analysis (WGCNA) was performed. Data information: *$p < 0.01$ by WGCNA for significant covariance by Pearson correlation, and blank squares are not significant.
